# Dyslipidemia in Obesity: Mechanisms and Potential Targets

**DOI:** 10.3390/nu5041218

**Published:** 2013-04-12

**Authors:** Boudewijn Klop, Jan Willem F. Elte, Manuel Castro Cabezas

**Affiliations:** Department of Internal Medicine, Diabetes and Vascular Centre, Sint Franciscus Gasthuis, Rotterdam, P.O. Box 10900, 3004 BA, The Netherlands; E-Mails: b.klop@sfg.nl (B.K.); jwfelte@planet.nl (J.W.F.E.)

**Keywords:** free fatty acid, postprandial lipemia, apolipoprotein B, non-HDL-C, small dense LDL, acylation-stimulation protein, statin, fibrate

## Abstract

Obesity has become a major worldwide health problem. In every single country in the world, the incidence of obesity is rising continuously and therefore, the associated morbidity, mortality and both medical and economical costs are expected to increase as well. The majority of these complications are related to co-morbid conditions that include coronary artery disease, hypertension, type 2 diabetes mellitus, respiratory disorders and dyslipidemia. Obesity increases cardiovascular risk through risk factors such as increased fasting plasma triglycerides, high LDL cholesterol, low HDL cholesterol, elevated blood glucose and insulin levels and high blood pressure. Novel lipid dependent, metabolic risk factors associated to obesity are the presence of the small dense LDL phenotype, postprandial hyperlipidemia with accumulation of atherogenic remnants and hepatic overproduction of apoB containing lipoproteins. All these lipid abnormalities are typical features of the metabolic syndrome and may be associated to a pro-inflammatory gradient which in part may originate in the adipose tissue itself and directly affect the endothelium. An important link between obesity, the metabolic syndrome and dyslipidemia, seems to be the development of insulin resistance in peripheral tissues leading to an enhanced hepatic flux of fatty acids from dietary sources, intravascular lipolysis and from adipose tissue resistant to the antilipolytic effects of insulin. The current review will focus on these aspects of lipid metabolism in obesity and potential interventions to treat the obesity related dyslipidemia.

## 1. Introduction

Obesity has turned into a worldwide epidemic. In the last decades the number of obese patients has increased considerably. It is especially alarming that in recent years the increase was most pronounced in children and that it occurs both in developed, but perhaps even more, in developing countries [1]. Visceral obesity leads to insulin resistance in part mediated by adipokines and free fatty acids (FFA). Adipokines such as resistin and retinol-binding protein 4 decrease insulin sensitivity, whereas leptin and adiponectin have the opposite effect. In addition, cytokines like TNF-α and IL-6, which originate from macrophages in adipose tissue, are involved [2]. Obesity, especially central obesity, is probably the main cause of the metabolic syndrome (MetS), which includes insulin resistance, type 2 diabetes mellitus, hypertension, the obstructive sleep apnea syndrome, non-alcoholic fatty liver disease (NAFLD) and dyslipidemia, all risk factors for cardiovascular disease [3,4]. Although doubts have arisen about the significance of the term metabolic syndrome in relation to cardiovascular complications, it has been suggested that identifying the condition will stimulate the physician to search also for the other risk factors clustering in the MetS [5].

The typical dyslipidemia of obesity consists of increased triglycerides (TG) and FFA, decreased HDL-C with HDL dysfunction and normal or slightly increased LDL-C with increased small dense LDL. The concentrations of plasma apolipoprotein (apo) B are also often increased, partly due to the hepatic overproduction of apo B containing lipoproteins [6,7]. The current review will focus on general lipid metabolism, the pathophysiological changes in lipid metabolism seen in obesity with the focus on postprandial lipemia and free fatty acid (FFA) dynamics and the potential pharmacological and non-pharmacological interventions.

## 2. Overview of Lipoprotein Metabolism

Numerous metabolic processes are involved in the uptake, transport and storage of lipids. After the ingestion of a meal containing fat, TG are lipolyzed in the intestinal lumen into FFA and 2-monoacylglycerols (MAG) and are taken up by the enterocytes via passive diffusion and specific transporters like CD36 [8]. Cholesterol is taken up by the enterocytes via the specific cholesterol transporter Niemann-Pick C1 Like 1 protein (NPC1L1) [9,10]. Once in the enterocyte, cholesterol is transformed into cholesterol-esters, whereas FFA and MAG are assembled into TG again. Finally, cholesterol-esters and TG are packed together with phospholipids and apolipoprotein (apo) B48 to form chylomicrons [8,11]. After assembly, the chylomicrons are secreted into the lymphatics and finally enter the circulation via the thoracic duct. The liver synthesizes TG-rich lipoproteins called very low density lipoproteins (VLDL), which increase postprandially when food derived TG and FFA reach the liver [11]. The assembly of VLDL is almost identical to the synthesis of chylomicrons, but apo B100 is the structural protein of VLDL (and its remnants, *i.e.*, intermediate density lipoproteins (IDL) and low density lipoproteins (LDL)) [11]. The human liver lacks the editing complex necessary to change the apo B100 molecule into the smaller apoB48, by post-transcriptional modification of one base leading to a premature stop codon [12].

Chylomicrons and VLDL deliver FFA to the heart, skeletal muscle and adipose tissue for energy expenditure and storage. Adequate lipolysis of TG-rich lipoproteins is necessary for FFA to be released in the circulation. This process is regulated by several enzymes and proteins acting as co-factors. Lipoprotein lipase (LPL) is the primary enzyme for TG lipolysis in the circulation and is strongly expressed in tissues that require large amounts of FFA like the heart, skeletal muscle and adipose tissue [13]. LPL serves as the docking station for chylomicrons and VLDL for adherence to the endothelium via glycosyl-phosphatidylinositol-anchored high-density-binding protein 1 (GPIHBP1), which is present on the luminal side of the endothelium [14,15,16]. The amount of liberated FFA from chylomicrons and VLDL depends on the activity of LPL, which is stimulated by insulin [17,18]. In contrast, apo C-III is an inhibitor of LPL, but also of hepatic lipase. Plasma apo C-III concentrations correlate positively with plasma TG [19]. In addition, chylomicrons compete with endogenous VLDL for the action of LPL [20]. The liberated FFA are avidly taken up by adipocytes and re-synthesized into TG within the cytoplasm where the acylation-stimulating protein (ASP)/C3adesArg pathway plays an important role [21,22]. The scavenger receptor CD36 is the best characterized FFA transporter and is abundant in muscle, adipose tissue and the capillary endothelium [23]. Insulin and muscle contractions increase the CD36 expression thereby facilitating FFA uptake [13].

The postprandial rise in insulin is one of the most important regulatory mechanisms for fuel storage. The postprandial increase of insulin results in the effective inhibition of hormone sensitive lipase, which is the key enzyme for hydrolysis of intracellular lipids. Despite the uptake of FFA by adipocytes and myocytes, a proportion of FFA remains in the plasma compartment (“spill over”) where the FFA are bound by albumin and transported to the liver [24]. When delivery of FFA for energy expenditure is insufficient like in the fasting state, FFA can be mobilized by adipose tissue for oxidation in energy demanding tissues like cardio myocytes. Insulin is also an important regulator of FFA mobilization from adipose tissue [17]. Therefore, insulin resistance has a major impact on the metabolism of TG-rich lipoproteins and FFA.

Eventually, chylomicrons and VLDL shrink in diameter during the process of lipolysis to form chylomicron remnants and dense LDL, respectively. Chylomicron remnants are taken up by the liver via multiple pathways including apo E, hepatic lipase, the LDL receptor, the LDL receptor-related protein and heparan sulphate proteoglycans [25,26,27,28,29,30]. In contrast, LDL is primarily taken up by the liver via the LDL receptor [31,32]. The LDL receptor is recycled and re-shuttled back to the cell surface. In the last decade, many studies have extended our knowledge concerning this recycling process of the LDL receptor, which is regulated by the proprotein convertase subtilisin/kexin type 9 (PCSK9) [32,33]. The LDL receptor undergoes lysosomal degradation during the shuttling process when PCSK9 is bound to the LDL receptor, but is recycled back to the surface of the hepatocytes in the absence of PCSK9 [33]. Neutralization of PCSK9 increases the total LDL binding capacity of the hepatocytes leading to reduced LDL-C concentrations [33].

Besides the above described TG and LDL metabolism, the intestine and liver also play an important role in the reverse cholesterol transport by the synthesis of HDL particles. HDL promotes the uptake of cholesterol from peripheral tissues, including the arterial wall, and returns cholesterol to the liver. Enterocytes and hepatocytes synthesize apo A-I which is the structural protein of HDL. Nascent HDL particles acquire free cholesterol from peripheral tissues. Subsequently, the cholesterol within HDL becomes esterified into cholesterol-esters by HDL associated lecithin-cholesterol acyltransferase (LCAT) [23]. Within the circulation, the HDL particles also become enriched with cholesterol-esters by the action of cholesterylester-transfer-protein (CETP) and phospholipid transfer protein (PLTP). In this process HDL acquires TG from TG-rich lipoproteins in exchange for cholesterol-esters as a direct consequence of the CETP action [11]. In the liver, hepatic lipase hydrolyses HDL-associated TG and also phospholipids inducing the formation of smaller HDL particles which can contribute again to the reverse cholesterol transport. Therefore, lipid metabolism is highly dynamic and depends on numerous factors including the postprandial state, TG-rich lipoprotein concentrations, HDL levels and function, energy expenditure, insulin levels and sensitivity and adipose tissue function.

## 3. Obesity Induced Changes in Lipoprotein Metabolism and Atherogenic Effects

**Figure 1:**
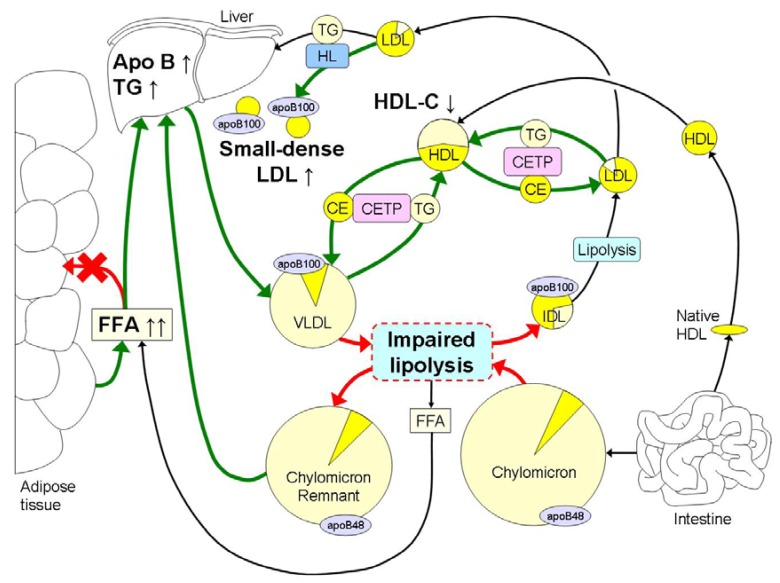
The hallmark of dyslipidemia in obesity is hypertriglyceridemia in part due to increased free fatty acid (FFA) fluxes to the liver, which leads to hepatic accumulation of triglycerides (TG). This leads to an increased hepatic synthesis of large very low density lipoproteins (VLDL) 1, which hampers the lipolysis of chylomicrons due to competition mainly at the level of lipoprotein lipase (LPL) with increased remnant TG being transported to the liver. Lipolysis is further impaired in obesity by reduced mRNA expression levels of LPL in adipose tissue and reduced LPL activity in skeletal muscle. Hypertriglyceridemia further induces an increased exchange of cholesterolesters (CE) and TG between VLDL and HDL and low density lipoproteins (LDL) by cholesterylester-transfer-protein (CETP). This leads to decreased HDL-C concentrations and a reduction in TG content in LDL. In addition, hepatic lipase (HL) removes TG and phospholipids from LDL for the final formation of TG-depleted small dense LDL. The intense yellow color represents cholesterol, whereas the light yellow color represents the TG content within the different lipoproteins. Obesity induced increases in metabolic processes are marked with green arrows, whereas reductions are marked with red arrows.

The hallmark of dyslipidemia in obesity is elevated fasting and postprandial TG in combination with the preponderance of small dense LDL and low HDL-C (Figure 1). Hypertriglyceridemia may be the major cause of the other lipid abnormalities since it will lead to delayed clearance of the TG-rich lipoproteins [34,35,36,37,38,39,40,41,42,43,44,45,46,47,48] and formation of small dense LDL [48,49].

Lipolysis of TG-rich lipoproteins is impaired in obesity by reduced mRNA expression levels of LPL in adipose tissue [50], reductions in LPL activity in skeletal muscle and competition for lipolysis between VLDL and chylomicrons [11]. Increased postprandial lipemia leads to elevated levels of FFA, resulting in detachment of LPL from its endothelial surface [51,52]. LPL may remain attached to VLDL and IDL contributing to further TG depletion. The exchange of TG from these remnants for cholesterol-esters from HDL by CETP with the concerted action of hepatic lipase, ultimately leads to the formation of small dense LDL [48,49]. In the presence of hypertriglyceridemia, the cholesterol-ester content of LDL decreases, whereas the TG content of LDL increases by the activity of CETP. However, the increased TG content within the LDL is hydrolyzed by hepatic lipase, which leads to the formation of small, dense LDL particles. The development of small dense LDL in obesity is mainly due to increased TG concentrations and does not depend on total body fat mass [53]. Small dense LDL are relatively slowly metabolized with a five day residence time, which enhances its atherogenicity [54].

Chylomicron remnants and LDL may migrate into the sub-endothelium and become trapped in the sub-endothelial space where they can be taken up by monocytes/macrophages [55,56,57]. Small dense LDL have an increased affinity for arterial proteoglycans resulting in enhanced subendothelial lipoprotein retention [58]. However, subendothelial remnants of chylomicrons and VLDL do not need to become modified to allow uptake by scavenger receptors of macrophages in contrast to native LDL [59]. It has been described that small dense LDL are more susceptible for oxidation, in part due to less free cholesterol and anti-oxidative content [60]. It should be noted that the lipoprotein size is a limiting factor for migration through the endothelium and that LDL particles migrate more easily than chylomicron remnants, but the number of migrated particles does not necessarily translate into more cholesterol deposition since chylomicron remnants contain approximately 40 times more cholesterol per particle than LDL [57]. Alternatively, LPL-enriched remnants of chylomicrons and VLDL may be transported to the tissues where interaction with proteoglycans and lipoprotein receptors lead to particle removal. This process takes place at the liver and acts as an anti-atherogenic mechanism, but it may also take place in other tissues where cholesterol can not be removed efficiently leading to cholesterol accumulation and therefore the initiation of the atherosclerotic plaque [56,57,61,62].

Studies using stable isotopes have shown a decreased catabolism of chylomicron remnants in obese subjects with the waist/hip ratio as best predictor for the fractional catabolic rate [63]. Taskinen and co-workers showed that the defective clearance of remnant lipoproteins can be explained by elevated concentrations of apo C-III in the situation of obesity [64]. Elevated levels of apo C-III in obesity can be explained by glucose-stimulated transcription of apo C-III and it has been described that plasma apo C-III levels correlate with fasting glucose and glucose excursion after an oral glucose test in obese humans [65]. Finally, the LDL receptor expression is reduced in obesity [66].

Remnants of chylomicrons and VLDL are involved in the development of atherosclerosis [67]. Several investigators have demonstrated an association between TG-rich lipoproteins and remnant cholesterol levels with the presence of coronary [34,35,36,38,39,40,41,42,68], cerebral [37], and peripheral atherosclerosis [69]. In addition to a direct detrimental effect by chylomicron remnants on vessels [59], impaired endothelial function after an oral fat load [70] and after infusion of artificial TG-rich lipoproteins have been described [71]. This phenomenon may take place by elevated levels of FFA [72], which are generated by the action of LPL mediated lipolysis. Other mechanisms of remnant-mediated atherogenesis which may play a role in obesity comprise the postprandial activation of leukocytes, generation of oxidative stress and production of cytokines [55,73,74].

Postprandial hyperlipidemia with accumulation of atherogenic remnants is especially linked to visceral obesity [75,76]. Postprandial lipid metabolism has been investigated in metabolic ward studies using non-physiological high amounts of fat [77]. A more physiological method to study postprandial lipemia has been developed in our laboratory, namely the measurement of daytime capillary TG profiles using repeated capillary self-measurements in an out of hospital situation [78,79]. It has been shown that diurnal triglyceridemia in obese subjects correlates better to waist circumference than to body mass index [78,80], which is in agreement with the hypothesis that the distribution of adipose tissue modulates postprandial lipemia [81]. All these mechanisms have been related to the higher incidence of cardiovascular disease seen in obesity [82].

HDL metabolism is also strongly affected by obesity because of the increased number of remnants of chylomicrons and VLDL together with impaired lipolysis. The increased number of TG-rich lipoproteins results in increased CETP activity, which exchanges cholesterolesters from HDL for TG from VLDL and LDL [60]. Moreover, lipolysis of these TG-rich HDL occurs by hepatic lipase resulting in small HDL with a reduced affinity for apo A-I, which leads to dissociation of apo A-I from HDL. This will ultimately lead to lower levels of HDL-C and a reduction in circulating HDL particles with impairment of reversed cholesterol transport [83].

## 4. Interplay between FFA Metabolism and Inflammation in Obesity: Crossroad between Innate Immunity and Lipid Metabolism

There are only two sources where plasma FFA may be derived from: firstly, lipolysis of TG-rich lipoproteins within the circulation and secondly, intracellular lipolysis in adipose tissue. An excellent review from the Oxford group described the relationship between plasma concentrations of FFA and insulin resistance as seen in obesity [17]. Other reviews have been published recently by several other groups as well [3,84]. It is widely recognized that plasma FFA are elevated in obese people as a consequence of an increased fatty acid release from adipose tissue and a reduction in plasma FFA clearance [85,86,87]. The increase in FFA and obesity-induced inflammation play a crucial role in the development of insulin resistance [88].

Various fatty acids are cytotoxic and their cytotoxicity depend on the type and has been extensively reviewed elsewhere [89,90]. Saturated fatty acids (SFA), arachidonic acid and linoleic acid (both polyunsaturated fatty acids (PUFA)) can mediate a diet-induced inflammation, although the literature concerning PUFA and inflammation is not consistent [89,90]. SFA, arachidonic acid and linoleic acid can stimulate the synthesis of pro-inflammatory cytokines like IL-1, IL-6 and TNF-α, whereas eicosepantenoic acid, a fish oil, has anti-inflammatory properties [89,90,91]. Since various fatty acids are cytotoxic, an “escape mechanism” should be present in order to remove FFA from the micro-environment where they are formed. In this process both, insulin and the acylation-stimulating protein (ASP)/C3adesArg-pathway play an important role in peripheral fatty acid trapping.

**Figure 2:**
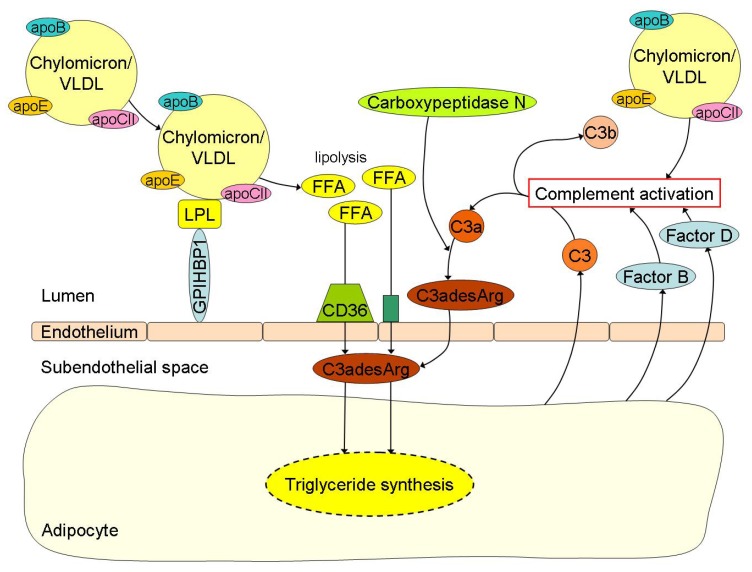
Free fatty acid (FFA) uptake and its related triglyceride (TG) synthesis in adipocytes are highly depended of C3adesArg or acylation-stimulation protein (ASP). Chylomicrons and VLDL undergo lipolysis by lipoprotein lipase (LPL) with subsequent release of FFA into the circulation. The FFA are then transported into the subendothelial space by the scavenger receptor CD36 and other transporters where C3adesArg plays an important role in the subsequent TG synthesis for storage of lipids in the adipocytes. C3adesArg is the most potent molecule known, which induces transmembrane transport of FFA and its intracellular esterification into TG within adipocytes. C3adesArg is metabolized from complement component (C) 3a by carboxypeptidase N and C3a is again the splice product from C3, which is formed in case of complement activation. Postprandial lipemia is directly linked to complement activation. For example, adipocytes secrete C3 when incubated with TG-rich lipoproteins like chylomicrons or very low density lipoproteins (VLDL), but also Factor B and Factor D, thereby causing activation of the complement cascade.

ASP in relation to peripheral fatty acid trapping was first described by Sniderman and collaborators [92]. In reaction to fatty acid delivery, adipocytes and fibroblasts secrete complement component 3 (C3) [93,94,95,96]. By the action of factor B and factor D (also secreted by adipocytes and fibroblasts) a small active fragment is split (C3a) from C3, which is readily converted into C3adesArg (also known as ASP) by carboxypeptidase N (Figure 2) [97]. C3adesArg, while not immunological active, has an important physiological role in the storage of fatty acids in adipocytes and other peripheral cells. Besides insulin, C3adesArg induces trans-membrane transport of fatty acids and their intracellular esterification into TG [21,22]. Recently, it has been described that ASP mRNA expression in visceral adipose tissue is reduced by approximately 40% in obese and morbidly obese subjects with or without insulin resistance when compared to lean controls [50]. In addition, C3adesArg mediates insulin-independent trans-membrane glucose transport [98]. It should be mentioned that these ASP-mediated processes only take place at peripheral cells and not in the liver. Fatty acid and glucose uptake by hepatocytes is ASP-independent.

In line with this ASP/C3adesArg concept are several studies, which investigated the role of the complement system in lipoprotein metabolism. Our group and others were able to demonstrate that the complement component 3 (C3) is one of the major determinants of the MetS [99,100,101] and postprandial lipemia in insulin resistant subjects, but also in insulin sensitive subjects [87,102,103]. C3 has also been genetically linked to the MetS in a recent meta-analysis of multiple genome wide association studies [104]. It was also demonstrated that a different component of the complement system, mannose binding lectin, may be involved in normal handling of postprandial lipoproteins [105]. Therefore, there is sufficient evidence supporting the notion that the complement system is an important regulator of postprandial fatty acid and TG metabolism and substantiates the concept that ASP/C3adesArg resistance plays a role in adequate peripheral fatty acid handling [102,106,107].

One of the difficulties in the evaluation of fatty acid metabolism is the determination of exact kinetics and trafficking of fatty acids between different tissues. Recent work from the Oxford group using arterio-venous blood sampling in adipose tissue with labeled palmitate, elegantly demonstrated impaired fatty acid trapping *in vivo* in obese men [18]. In addition, treatment of insulin resistance with metformin has been shown to reduce plasma FFA concentrations by lowering fasting FFA levels but without any effect on catecholamine mediated lipolysis of adipocytes [108]. Moreover, obese men also showed decreased uptake of dietary fat by adipose tissue, which results in a higher delivery of chylomicron remnants to the liver with consequently enhanced VLDL-TG being delivered to peripheral adipocytes [18]. The authors referred to this situation as “a seemingly unnecessary loop of fatty acid trafficking to the liver” and associated that to increased liver fat content. 

## 5. Lifestyle Interventions for Dyslipidemia in Obesity

Treatment of obesity-associated dyslipidemia should be focused on lifestyle changes including weight loss, physical exercise and a healthy diet. Lifestyle changes synergistically improve insulin resistance and dyslipidemia [59]. The amount of ingested fat and total calories are the most important dietary factors to induce obesity and its related postprandial lipemia [109]. This has already been demonstrated in early childhood [110]. Weight loss has been demonstrated to markedly reduce fasting and non-fasting TG concentrations, which can be attributed to an increase in LPL activity with a concomitant reduction in apo C-III levels [111], a decrease in CETP activity [112,113] and an increased catabolism of TG-rich lipoproteins [114]. Besides reductions in fasting and non-fasting TG concentrations, a small reduction in LDL-C can be expected upon weight loss, which may be attributed to increased LDL receptor activity. A weight loss of 4–10 kg in obese subjects resulted in a 12% reduction in LDL-C and a 27% increase in LDL receptor mRNA levels [111,115].

The type of dietary fat also affects postprandial lipemia [109]. A study in rats showed that a diet high in saturated fats reduced LPL protein levels and LPL activity in skeletal muscle, whereas LPL activity was increased in adipose tissue favoring shunting of lipids from skeletal muscle to adipose tissue [116]. Moderate weight loss (approximately 10%) in obese, but otherwise healthy men, which was induced by a diet low on carbohydrates and SFA and high on mono-unsaturated fatty acids (MUFA) resulted in a 27%–46% reduction in postprandial TG levels [117]. Long term intervention with MUFA resulted in a reduction in postprandial inflammation when compared to a diet rich in SFA in patients with the MetS [118]. 

Recent genome wide association studies have found more than 95 loci associated with lipid levels, but together they explain less than 10% of the variation in lipids. Interactions between genes, obesity and lipid levels but also with the type of dietary fat consumed have recently been described [119,120,121,122]. Homozygosity for the C allele of the APOA2 −265T > C polymorphism was associated with an increased obesity prevalence compared to the TT + TC genotype in those subjects with high SFA consumption (OR 1.84 95% CI 1.38–2.47) [120]. In a Spanish population with a relatively high MUFA intake, carriers of the minor C allele of the APOA5 −1131T > C polymorphism, which is associated with increased plasma TG, appear to be more resistant to weight gain by fat consumption and showed an inverse relationship between fat intake and plasma TG [122]. However, high PUFA consumption was associated with increased plasma TG and decreased LDL particle size in carriers of the C allele in a U.S. population [121]. These results suggest the potential usefulness of a nutrigenomic approach for dietary interventions to prevent or treat obesity and its related dyslipidemia.

Physical exercise has been shown to increase LPL and hepatic lipase activity, which stimulates TG lipolysis [123,124]. The mechanism of exercise-induced LPL activity remains unclear, but it was hypothesized that exercise stimulates especially muscular LPL activity, although this could not be confirmed in a recent study [125]. A 12-week walking program supplemented with fish oil (1000 mg eicosepantenoic acid and 700 mg docosahexaenoic acid daily) in subjects with the MetS resulted in lower fasting TG and decreased the postprandial response of TG and apoB48 [126]. Exercise training for 16 weeks in obese subjects with NAFLD resulted in a small reduction in intra-hepatic TG content, although no changes in VLDL-TG or apoB100 secretion were observed [127]. Exercise induced reductions in intra-hepatic TG content have also been reported even in the absence of weight loss [128]. Moreover, intra-hepatic TG content was reduced in overweight men after a low fat diet for three weeks, whereas a high fat diet increased intra-hepatic TG [129]. The plasma TG lowering effect of exercise and weight loss is the most consistent finding in studies concerning blood lipids [130], whereas increasing HDL-C levels by exercise remains controversial, especially in those subjects with high TG and low HDL-C levels [131].

Other dietary factors besides calorie restriction and the type of dietary fat have also been shown to have beneficial effects on dyslipidemia. Dietary intake of resistant starch, a dietary fiber, has been shown to improve nutrient absorption and has also been linked to insulin metabolism. Daily intake of resistant starch from bread, cereals, vegetables and pastas is approximately 5 g/day in the Western world, which is highly insufficient for potential health benefits [132]. Recently, a randomized study in 15 insulin resistant subjects has shown that 8 weeks of resistant starch supplementation (40 g/day) improved insulin resistance and subsequently FFA metabolism. Resistant starch ingestion resulted in lower fasting FFA concentrations, increased TG lipolysis by enhanced expression of related genes like LPL together with increased FFA uptake by skeletal muscle [133]. However, no effect from resistant starch supplementation was observed on TG and cholesterol concentrations [132,133].

Unfortunately, lifestyle modifications are often insufficient to achieve weight loss and improvement of the dyslipidemia. A recent meta-analysis concerning anti-obesity drugs reported a mean weight loss of 3.13 kg, but marked improvements in dyslipidemia were absent [134]. Orlistat, which reduces the lipolysis of TG within the gastrointestinal system and thus prevents absorption of intestinal fat by 30%, showed only a modest reduction in LDL-C of 0.21 mmol/L. Sibutramine, which increases the sensation of satiety by modulating the central nervous system, showed a 0.13 mmol/L reduction in TG, whereas rimonabant did not show any lipid improvements [134]. Finally, bariatric surgery-induced weight loss has been associated with decreased TG and increased HDL-C levels [135].

## 6. Lipid Targets and the Pharmacological Treatment of Dyslipidemia in Obesity

The EAS/ESC guidelines recommend to profile lipids in obese subjects in order to assess cardiovascular risk [136]. However, the necessity to initiate pharmacological treatment next to lifestyle intervention in obese subjects with dyslipidemia depends on co-morbidity, the potential underlying primary lipid disorders and the calculated cardiovascular risk [11,136]. High risk subjects with primary lipid disorders like familial hypercholesterolemia or familial combined hyperlipidemia as well as subjects with known diabetes mellitus or cardiovascular disease all require appropriate pharmacological treatment independent from obesity [136,137]. Nevertheless, the presence of obesity can affect treatment targets since obesity may contribute to increased remnant cholesterol, higher TG levels and lower HDL-C concentrations. Therefore, apo B or non-HDL-C levels are recommended as secondary treatment targets next to LDL-C levels in the presence of the hypertriglyceridemic waist [11,136,138]. Apo B represents the total *number* of atherogenic particles (chylomicrons, chylomicron remnants, VLDL, IDL and LDL), whereas non-HDL-C represents the *amount of cholesterol* in both the TG-rich lipoproteins and LDL. Recently, a meta-analysis has shown that implementation of non-HDL-C or apo B as treatment target over LDL-C would prevent an additional 300,000–500,000 cardiovascular events in the US population over a 10-year period [139]. Although others did not describe any benefit of apo B or non-HDL-C over LDL-C levels to assess cardiovascular risk [140,141,142]. The treatment target for non-HDL-C should be 0.8 mmol/L higher than the target for LDL-C, which corresponds with non-HDL-C levels of 3.8 mmol/L and 3.3 mmol/L for subjects at moderate and high risk, respectively. Treatment targets for apo B are approximately 0.80–1.00 g/L [136]. Specific treatment targets for TG levels are unavailable, especially since TG are highly variable and increase during the day [143]. However, pharmacological interventions to lower specifically TG should be initiated when TG exceed 10 mmol/L to reduce the risk for pancreatitis [11,144]. In addition, additional diagnostic tests are warranted to test for the presence of familial hypertriglyceridemia or familial dysbetalipoproteinemia [11,136,138,144].

Statins are the first choice drug of all pharmacological agents to reduce LDL-C, non-HDL-C and/or apo B. However, statins lower TG only marginally and do not fully correct the characteristic dyslipidemia seen in obesity, which may contribute to the residual risk after initiating statin therapy [145]. Statins inhibit the enzyme 3-hydroxy-3-methylglutaryl-coenzyme A (HMG-CoA), which is the rate limiting step in the hepatic cholesterol synthesis. This efficiently increases the fractional catabolic rate of VLDL and LDL together with a slight reduction in hepatic secretion of VLDL. Therefore, statins lower both remnant cholesterol and LDL-C levels [146].

Recently, strategies for combination therapies with statins to achieve even lower cholesterol levels have been reviewed [145,146,147,148,149,150]. Combinations can be made with ezetimibe, which inhibits the intestinal cholesterol absorption by interaction with NPC1L1, which results in an additional 20% lowering effect on LDL-C, but without affecting TG or HDL-C concentrations. On the contrary, fibrates are primarily indicated in the case of hypertriglyceridemia and they reduce TG by approximately 30% and LDL-C by 8%, whereas HDL-C is increased by an average of 9% [149]. Fibrates (fibric acid derivatives) are peroxisome proliferator-activated receptor-α agonists, which transcriptionally regulate lipid metabolism related genes. Fibrates as monotherapy have been shown to reduce cardiovascular mortality, especially in subjects with characteristics of the MetS with TG levels > 2.20 mmol/L [151,152,153,154,155]. However, there is controversy about the effectiveness of fibrate therapy on top of statin therapy since the ACCORD trial was unable to confirm a beneficial effect on cardiovascular endpoints by fenofibrate combined with statins in diabetic patients [156]. Although subgroup analyses suggested a beneficial effect from combination therapy of fibrates with statins in patients with diabetes and the characteristic dyslipidemia with high TG and low HDL-C [156]. Therefore, fenofibrate may be used to treat residual dyslipidemia in diabetic patients on top of statin therapy [145].

Nicotinic acid inhibits the lipolysis of adipocytes, which results in decreased FFA levels, reduced VLDL synthesis, a slight increase in HDL production rate and decreased catabolism of HDL [146]. These changes by niacin subsequently lead to 15%–35% lower TG levels and 10%–25% higher HDL-C concentrations [11,146]. Recently, it has been shown that the addition of niacin to patients with a known history of cardiovascular disease, typical dyslipidemia and intensively controlled LDL-C levels with statin therapy did not lead to clinical benefit despite a reduction in fasting TG and increase in HDL-C [157]. However, specific data concerning combination therapy of niacin with statins in obesity remains scarce. Omega-3 fatty acids, which decrease the hepatic synthesis and accumulation of TG [158], have been shown to reduce plasma TG by 25%–30% by effectively reducing the hepatic secretion of VLDL in insulin resistant subjects [146,159]. Omega-3 fatty acids have also been shown to increase the conversion of VLDL into IDL, which suggests an additional benefit for combining omega-3 fatty acids with statins by increased catabolism of VLDL, IDL and LDL [159].

Drugs that increase insulin sensitivity like metformin or thiazolidinedione derivatives, have no [108] or minimal effects on the lipoprotein profile in obesity [160]. In the case of thiazolidinedione derivatives, their mode of action causes an increase of body weight, due to expansion of the subcutaneous fat compartment, which makes these drugs less appropriate in the case of obesity [160].

## 7. Conclusions

The pathophysiology of the typical dyslipidemia observed in obesity is multifactorial and includes hepatic overproduction of VLDL, decreased circulating TG lipolysis and impaired peripheral FFA trapping, increased FFA fluxes from adipocytes to the liver and other tissues and the formation of small dense LDL. Impairment of the ASP/C3adesArg pathway probably contributes to the typical dyslipidemia as well. Treatment should be aimed at weight loss by increased exercise and improved dietary habits with a reduction in total calorie intake and reduced SFA intake. Medical therapy can be initiated if lifestyle changes are insufficient. Statins are the primary lipid lowering drugs with effective reductions in LDL and remnant cholesterol levels. Moreover, the addition of fibrates may be considered in case of residual dyslipidemia in subjects with diabetes mellitus, elevated TG and reduced HDL-C levels. ApoB and/or non-HDL-C concentrations reflect the atherogenic lipid burden more accurately than LDL-C alone in obesity and should be used as treatment targets.
